# Automatic segmentation of ablation lesions and termination of the image acquisition/analysis process

**DOI:** 10.1186/1532-429X-13-S1-P245

**Published:** 2011-02-02

**Authors:** Andriy V Shmatukha, Eugene Crystal

**Affiliations:** 1 Cardiac and Interventional Applied Science Laboratory, General Electric Healthcare, Toronto, ON, Canada; 2 Arrhythmia Services, Schulich Heart Centre, Sunnybrook Health Sciences Centre, Toronto, ON, Canada

## Objective

Automate the processes of ablation lesion imaging and delineation in order to make them non-expert user friendly.

## Background

Visualization of radiofrequency ablation lesions during cardiac electrophysiology procedures would help ensuring their contiguity and inclusiveness, which are essential for the procedures’ long-term success.

The usefulness of dynamic contrast enhancement (DynCE) and cumulative characteristics for ablation lesion visualization has been already demonstrated (1). However, planning of the image acquisition process and interpretation of the resulting images may pose a challenge for electrophysiologists who don’t interpret MRI routinely.

We describe an algorithm allowing automatic discrimination between ablation lesions and surrounding normal tissue during DynCE scans as well automatic termination of the image acquisition and analysis processes as soon as the desired lesion visibility level has been achieved.

## Methods

56 lesions were ablated in the Latissimus dorsi muscles of 15 rabbits using clinical catheters and time/power settings. The animals underwent MRI at various times after ablations using various imaging techniques. DynCE images were post-processed using original algorithms and software (1).

## Results

**Figure 1:**
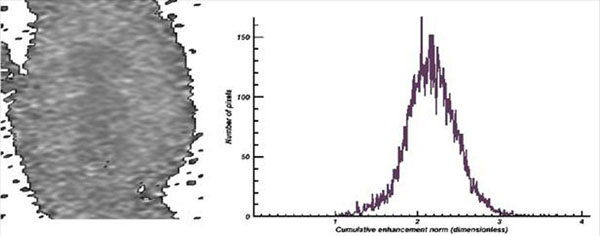
Cumulative Enhancement Norm (CEN) map at 21 sec. after contrast agent (CA) injection (left) and its histogram (right). Actual map size: 110 x 59 mm (176 x 86 px)

**Figure 2:**
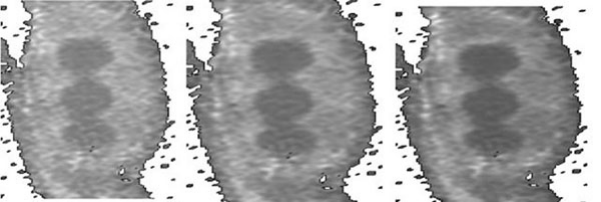
CEN maps at 83 (left), 167 (middle) and 313 (right) sec. after CA injection. The same CynCE data set as on Fig 1.

**Figure 3:**
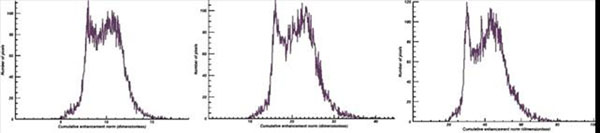
Histogram of the CEN maps depicted on Figure 2: 83 (left), 167 (middle) and 313 (right) sec. after CA injection.

**Figure 4:**
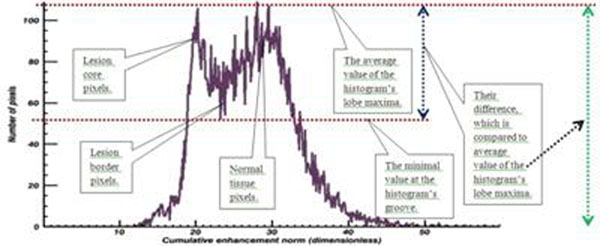


Lesion non-detectability on early contrast agent wash-in cumulative DynCE images strongly correlated with lack of lesion and normal tissue separation on their histograms (Fig. 1). As wash-in continued and new data was acquired and post-processed, ablation lesions became more apparent (Fig. 2) and separated from normal tissue on histograms (Fig. 3): lower-intensity histogram peaks were formed by lesion core pixels, higher-intensity peaks were formed by normal tissue pixels, and lesion border pixels composed the groove segment between these peaks (Fig. 4).

**Figure 5:**
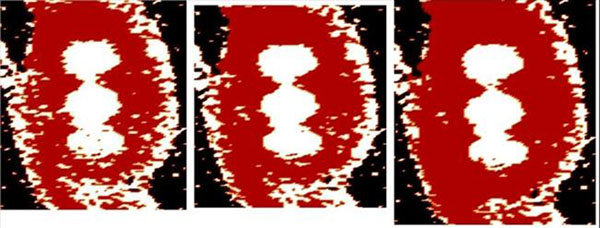
Segmented (from CEN maps) images depicting the normally enhancing pixels in red, non-enhancing (perfusion deficient) ones in white, and empty spaces on the original image in black. Shown at 83 (left), 167 (middle) and 313 (right) sec. after CA injection. Actual image size: 110 x 59 mm (176 x 86 px). The same DunCE data set as on the figures above.

**Figure 6:**
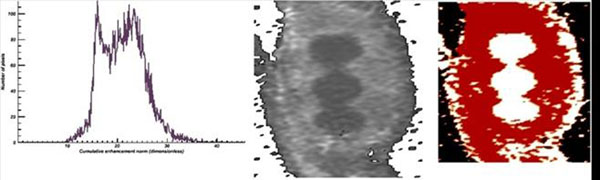
The histogram as well as CEN and lesion segmentation map at the “final” dynamic – 167 sec. after CA injection and 146 sec. before the end of the actual data acquisition.

**Figure 7:**
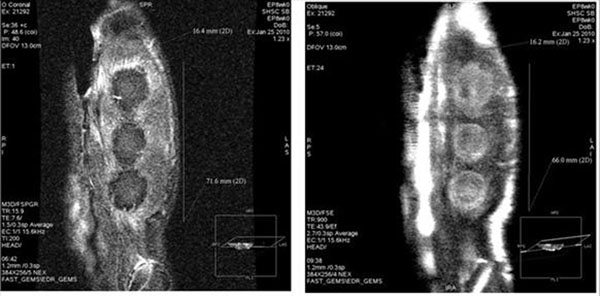
3D delayed enhancement (left) and T2w FSE (right) images acquired in the same animal model during the same experiment (as the DynCE images mentioned herein) reformattd to be approximately in the same plane as the rest of the images.

Our algorithm automatically identified the peaks and groove, and used the information to discriminate between actively and poorly enhancing pixels (Fig. 5). It also compared the peaks’ values to the groove’s one and used the information to terminate image post-processing when satisfactory lesion-to-tissue contrast was detected (Fig. 6). The resulting segmented images demonstrated good correspondence to other lesion depicting MR images acquired during the study (Fig. 7).

## Conclusions

Our algorithm demonstrated a good performance in this study and has a potential to prove robust and useful in real clinical conditions. More accurate and noise-resistant histogram analysis and segmentation methods can be implemented, which would result in more robust ablation lesion delineation and the reduction of the DynCE scan time required for it. Automatic lesion detection and scan termination tools based upon this approach have a potential to ease and promote the acceptance of intra-procedural MRI by the clinical electrophysiologist.
